# Scharioth Macula Lens for patients with high myopia: a novel approach to achieve spectacle independence (binocular trifocal monovision)

**DOI:** 10.22336/rjo.2023.11

**Published:** 2023

**Authors:** Gabor Bernd Scharioth, Ali Duran, Hanga Béres

**Affiliations:** * Aurelios Augenzentrum Recklinghausen, Recklinghausen, Germany; ** Department of Ophthalmology, University of Szeged, Szeged, Hungary

## Abstract

We aimed to test a novel concept based on multiple IOL-implantation, targeting spectacle independence for patients with high and excessive myopia (26.0 mm < axial length; 6.0 D < refractive error). Therefore, we introduced the first results of five patients with high myopia. After clear lens extraction, one eye was targeted to emmetropia, and the other to mild myopia by implanting monofocal capsular bag IOLs with appropriate refractive powers in each case. The emmetropic eye was aimed to result in magnification and improved distance vision, while the mild myopic eye was supposed to ensure good intermediate vision. Thereafter, a Scharioth Macula Lens (SML) was implanted into the emmetropic eye in order to achieve sharp near vision. Visual acuity curves and defocus curves were plotted postoperatively.

According to our results, this new concept seems to be an efficient approach of achieving appropriate uncorrected vision at all distances, by creating binocular trifocal monovision.

## Introduction

Pathological myopia is a major cause of irreversible vision loss and is among the leading causes of blindness in the US and Japan [**1**]. It is well known that there is a higher prevalence of high myopia in Asian and a lower prevalence in African populations [**2**,**3**].

The progression of high myopia is associated with declining visual acuity, visual impairment and is also a powerful risk factor for cataract formation [**4**]. Choroidal neovascularization (CNV) develops in approximately 10% of eyes with degenerative myopia [**1**,**4**]. 

The treatment of high myopia is constantly continuing to challenge refractive surgeons. Traditional surgical interventions, such as refractive laser surgery techniques do not represent an optimal solution, as all of them are irreversible, and the clinical outcomes may be less predictable, than in cases of lower levels of myopia [**5**] Haze has been reported to be a significant long-term problem in eyes with high myopia treated with PRK [**5**]. Although, compared to PRK, femto-LASIK provides more favorable results in terms of visual outcomes, residual refractive error and contrast sensitivity, these cases showed a significant increase of ocular high order aberrations (HOA) and coma [**6**]. On the other hand, laser refractive surgery techniques can correct myopia only up to -12 to -14 diopters (D). As the higher intended correction needed, the thinner and flatter the cornea will be post-operatively. In cases of extreme myopia, the thickness of the stromal bed of the cornea is more likely to decrease under a critical 250-300 µM, which can consequently lead to further complications, such as corneal ectasia and decreased visual quality.

Phakic intraocular lenses, including implantable collamer lenses (ICL) have brought a remarkable progress in the management of high and extreme myopia, as compared to keratorefractive surgeries, being less likely to produce HOA and provide better outcomes of visual quality defined by modular transfer function (MTF), point spread function (PSF), visual acuity, and contrast sensitivity [**6**-**8**]. Nevertheless, changes in wavefront aberrations after the operation are significant, adverse impacts being the most obvious on near vision [**9**]. Reported complications and long-term safety concerns include endothelial cell loss, cataract formation, secondary glaucoma, iris atrophy and traumatic dislocation [**10**]. In addition, the lens power calculation and surgical implantation of phakic IOLs require special techniques [**5**]. 

Removal of the clear, not cataractous crystalline lens (clear lens extraction, CLE), or that for refractive purposes (refractive lens exchange; RLE) present several advantages over corneal refractive surgery, however, careful patient selection is essential, especially to avoid the most frequent complication, retinal detachment [**11**]. Cornea and the anterior segment of the eye remain essentially intact, and lens removal followed by the implantation of an optimal intraocular lens (e.g. multifocal or toric lenses) might correct all refractive errors including presbyopia and astigmatism, and eliminates the future need for cataract surgery [**11**]. The intraocular power calculation for CLE or RLE is similar to standard calculations used for cataract surgery, however, precise preoperative measurements and the application of IOL calculation formulae, which are also reliable in the case of long axial length (e.g. Haigis), are crucial. Nevertheless, exact calculation remains challenging, as the effective lens position (ELP) might be different in case of extreme power IOLs, compared to “regular” power lenses [**12**-**17**]. Calculations become even more complicated, if the patient had had previous corneal refractive surgery. The actual desired postoperative refraction should also be discussed, since a moderate myopia (-1.00 D) may be favorable in both eyes in case of monofocal IOL use [**11**]. Other surgeons prefer to target for myopic monovision.

As these patients are usually much younger than those requiring cataract surgery, the loss of lens accommodation should also be taken into consideration [**11**], especially because patients with high myopia are often willing to be made independent from the use of spectacles or contact lenses. Multifocal IOLs (MIOLs) primary used for presbyopia-correction might seem to be the most obvious choice, however, they should only be implanted in eyes with no ocular disorder; thus, a detailed preoperative retina examination is recommended to exclude progressive maculopathy [**15**]. The other limiting factor might be the lack of appropriate (low or negative) IOL powers on the market, especially in the case of extreme myopes [**15**]. With the most MIOLs available, postoperative refractive errors are common among myopic patients, and the addition offered by these lenses are often not enough for high myopes, therefore neither spectacle independence, nor visual comfort will be achieved. Moreover, higher addition MIOLs are more likely to cause visual side effects [**15**,**18**], and additionally, these IOLs implanted into large myopic eyes are also more prone to tilt and decentration [**17**,**19**].

## Description of the technique

According to the personal experience of the authors with high myopic patients in their praxis, they usually profit from lens extraction and emmetropization, due to the enlarged image in their distance vision, but often complain about the loss of near vision, and report that near vision with reading glasses is even worse, than that it used to be without spectacle correction, prior to surgery.

## The Scharioth Macula Lens (SML)

**Fig. 1:**
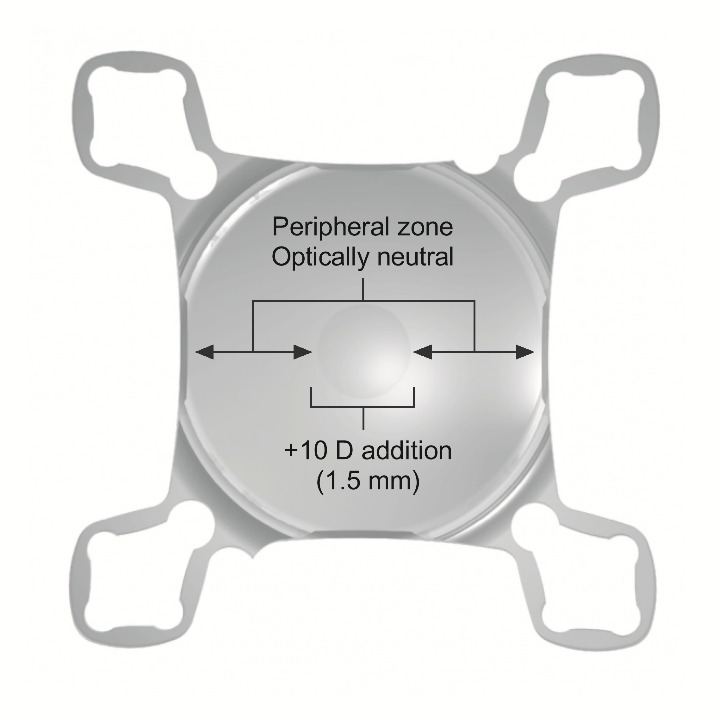
The Scharioth Macula Lens (SML) with the central 10.0 D addition, and refractive peripheral optical surface

The SML is an implantable intraocular magnifying device (a simple intraocular lens) primarily developed for pseudophakic AMD-patients [**20**], however, it was hypothesized that patients suffering from various diseases leading to macular degeneration might also benefit from SML-implantation (**Fig. 1**). SML uses the effect of near vision miosis, since the pupil constricts in a reflex when the eye focuses on a near object [**20**]. This reflex works reliably also in myopic people.

## The concept of Binocular Trifocal Monovision

After implanting the Scharioth Macula Lens (SML) over the past few years in myopic patients with myopic macular degeneration (MD), resulting in significant near vision improvement and high satisfaction of the patients, it was hypothesized that a new concept targeting spectacle independence could probably bring solution for patients with high and excessive myopia: briefly, after CLE, one eye will be targeted to emmetropia, and the other to mild myopia by implanting monofocal capsular bag IOLs with appropriate refractive powers. The emmetropic eye will result in magnification and improved distance vision, while the mild myopic eye could ensure good intermediate vision. During the same intervention (or anytime thereafter), a SML will be implanted into the ciliary sulcus of the emmetropic eye to achieve an excellent near vision. With this concept, a “Binocular Trifocal Monovision” could be accomplished and patient could be completely spectacle independent.

Based on this principle, the purpose of the current study was to evaluate the impact of this novel multiple IOL-implantation approach on the visual outcomes, reading ability, spectacle independence and satisfaction of five patients with high myopia, and depending on the results, to consider the further testing of the approach, and the possible extension of the indications for the use of the SML.

## Surgical technique

Five female patients between 48 and 71 years old with excessive myopia were enrolled in our pilot study. None of them had any history or symptoms of dry eye, any allergies of which they were aware, and they did not report any previous eye-related pathologies. A complete preoperative ophthalmologic examination was performed in each case. The most relevant biometric characteristics of each patient are presented in **Table 1**.

**Table 1:** Demography and biometry data of the five patients. Lengths are expressed in millimeters

Patient	Age	Sex	Dominant Eye	AXL		ACD		K1		K2	
				OD	OS	OD	OS	OD	OS	OD	OS
1	71	Female	OS	28.71	25.70	3.06	3.05	7.83	7.69	7.43	7.48
2	48	Female	OS	30.56	29.03	3.94	3.86	7.51	7.64	7.20	7.16
3	66	Female	OD	26.42	26.77	3.21	3.12	7.74	7.66	7.43	7.48
4	56	Female	OS	28.91	28.75	3.84	3.80	7.65	7.66	7.61	7.49
5	57	Female	OS	27.41	26.55	3.22	3.19	7.79	7.77	7.53	7.51
AXL = Axial Length (mm), ACD = Anterior Chamber Depth (mm), K1 = Keratometry 1 (mm), K2 = Keratometry 2 (mm), OD = Right eye, OS = Left eye.											

Visual acuity for distance was assessed by using ETDRS charts and stated in decimal format. Near vision testing and reading simulation tests with +2.5 D at 40 cm and with +6.0 D at 15 cm were performed by using the German version of the Radner reading chart (Radner Lesetafeln; Norbert Werner GmbH) [**21**]. Slit lamp examination and the measurement of intraocular pressure were performed during each visit.

As for CLE, the dominant eye of the patients was targeted to emmetropia in each case, while we aimed to achieve a residual refraction of -1.0 D in the case of the non-dominant eyes. Monofocal one-piece intraocular lenses (IOLs) were implanted through clear corneal incision into the capsular bag of both eyes (implanted models and IOL-powers are presented in **Table 2**) after a routine, uneventful clear lens extraction using the standard phacoemulsification method. Wounds were left sutureless in each case. The implantation of the primary IOL into both eyes was performed during the same surgical intervention in case of each patient, without any adverse events or complications related to either the surgery or the IOL-implantations. The immediate postoperative course was uneventful with all our patients.

**Table 2:** Primary capsular bag IOLs implanted into the patients’ eyes (*dominant eye)

Patient	Eye	Target refraction (D)	Primary IOL type	Primary IOL SEQ (D)
1	OD	0.0	J&J Sensar AR40	7.0
	OS*	0.0	J&J Sensar AR40	13.5
2	OD	0.0	J&J Sensar AR40M	-2.0
	OS*	-0.5	J&J Sensar AR40M	4.0
3	OD*	0.0	J&J Tecnis ZCT225 toric	11.5
	OS	-0.5	J&J Tecnis ZCT150 toric	11.0
4	OD	-1.0	Medicontur Bi-Flex 877PA	6.0
	OS*	-0.5	Medicontur Bi-Flex 877PA	5.0
5	OD	-1.0	Alcon AcrySof IQ SN60WF	10.5
	OS*	-0.3	Alcon AcrySof IQ SN60WF	11.5
D = Diopter, OD = Right eye, OS = Left eye				

The SML was implanted into the emmetropia-targeted eye of each patient. We performed the Radner reading test inherently developed for AMD-patients prior to the SML-implantation [**20**], following the recommended protocol: reading at 40 cm with the addition of 2.5 D, then reading at 15 cm with the addition of 6.0 D (simulating reading with the SML). All five patients achieved an improvement in near vision, hence, we concluded that all of them seemed to have been good candidates for the SML-implantation, and a significant improvement in near vision could be expected.

The SML-surgery was performed 3 weeks after the CLE in each case. The A45SML posterior chamber IOL by Medicontur (Medicontur Medical Engineering Ltd., Zsámbék, Hungary) was implanted into the ciliary sulcus of the dominant eye of the patient through a minimum of 2.2 mm clear corneal incision. Proper positioning of the haptics and the centration of all three IOLs were checked in each case. Finally, the OVD was removed and the incisions were hydrated to prevent leakage. Wound was left sutureless in all cases.

## Results

Postoperative visual assessments were performed one week and approximately one month after the SML-implantation. Two of the five patients could be followed up for one and one-and-half years, and we could obtain middle-term results (four months postop) from one subject.

**Table 3** underlines the spherical equivalent refraction (SEQ) based on subjective spherical and cylindrical errors of each patient measured preoperatively after the implantation of the capsular bag IOLs, and finally after the SML-implantation. SEQ results have decreased significantly in each eye compared to the preoperative values (p<0.0001; calculated with the paired t-test), and refractive outcomes remained essentially unchanged thereafter (p=0.9102).

**Table 3:** Subjective refractions measured preoperatively, after the implantation of the primary IOL, and after the SML implantation. Results with italic letters were obtained four months postoperatively

Patient	Eye	SPH				CYL				SEQ			
		Preop	After primary IOL	After SML (Month 1)	After SML (Year 1-1.5)	Preop	After primary IOL	After SML (Month 1)	After SML (Year 1-1.5)	Preop	After primary IOL	After SML (Month 1)	After SML (Year 1-1.5)
1	OD	-18.25	-0.50	-0.50	-0.50	-0.75	-1.25	-1.00	-1.50	-18.63	-1.125	-1.00	-1.25
	OS*	-8.25	-0.75	0.00	-0.25	-0.75	-0.25	-1.25	-1.50	-8.63	-0.875	-0.625	-1.00
2	OD	-18.0	1.00	1.50	1.50	-1.50	-2.00	-1.75	-1.50	-18.75	0.00	0.625	0.75
	OS*	-13.25	0.25	-1.50	-1.00	-1.75	-2.75	-2.25	-2.00	-14.13	-1.125	-2.625	-2.00
3	OD*	-6.00	0.50	0.00	*0.00*	-2.25	-0.50	-0.50	*-0.50*	-7.125	0.25	-0.25	*-0.25*
	OS	-7.75	0.00	-0.25	*0.00*	-1.50	-0.75	-0.75	*-0.50*	-8.50	-0.375	-0.625	*-0.25*
4	OD	-14.0	-0.75	-0.75	-0.75	-0.50	-0.25	-0.25	-0.50	-14.25	-0.875	-0.875	-1.00
	OS*	-13.25	0.50	0.25	0.25	-1.00	-1.25	-1.50	-1.25	-13.75	-0.125	-0.50	-0.375
5	OD	-10.0	-0.75	-0.50	-0.25	0.00	-0.75	-1.00	-1.25	-10.00	-1.125	-1.00	-0.875
	OS*	-7.50	0.25	-0.25	-0.50	-1.75	-1.00	-1.00	-1.50	-8.38	-0.25	-0.75	-1.25
Preop = Preoperatively, SML = Scharioth Macula Lens, SPH = Sphere, CYL = Cylindric refraction, SEQ = Spherical Equivalent Refraction, IOL = Intraocular Lens, OD = Right Eye, OS = Left eye													

**Table 4** presents the uncorrected and corrected distance and near visual acuities (UDVA, CDVA, UNVA, CNVA, respectively) measured preoperatively, after the implantation of the capsular bag IOLs, and one month after the implantation of the SML. Middle- and long-term results were also indicated where they were available. Uncorrected distance vision remained unaffected. CDVA improved significantly after the implantation of the capsular bag IOLs compared to the preoperative measurements (p=0.0078; calculated with the non-parametric Wilcoxon matched-pairs signed rank test), and visual outcomes remained unaffected thereafter (p=0.7500, and p>0.9999, respectively).

Prior to the implantation of the SML, CNVA was tested both with 2.5 D addition and reading from 40 cm, and with 6.0 D addition and reading from 15 cm. This latter setup offered the prediction of UNVAs after the SML-implantation. A significant improvement of near vision could be predicted in all eyes (p=0.0156). Actually, achieved UNVA in the SML-implanted dominant eyes showed a remarkable improvement in all five patients compared to the results measured with the 2.5 D addition prior to the SML-implantation (p=0.0154). One month postoperatively, uncorrected near visual outcomes were identical or even superior to the preoperative predictions (p=0.5000). From the fourth postoperative month on, all five patients were able to read A+ on the Radner reading chart without any further vision correction. As myopes are already used to reduced reading distance, it was not surprising, that the adaption to reading from 15 cm seemed to be easy for all patients, compared to the AMD-patients who were implanted the SML. IOP remained essentially unchanged during the whole surgical and follow-up period.

**Table 4:** Uncorrected and corrected distance and near visual acuities before and after the implantation of the primary IOL and the Scharioth Macula Lens. Results of the dominant eyes are indicated with bold letters. Distance visual acuities are expressed in decimal. Near visual acuities were determined using the Radner reading chart. Results with italic letters were obtained four months postoperatively

Patient	Eye	UDVA		CDVA				CNVA		UNVA	
		After SML (month 1)	After SML (Year 1-1.5)	Preop	After primary IOL	After SML (Month 1)	After SML (Year 1-1.5)	Addition 2.5 D	Addition 6.0 D	After SML (Month 1)	After SML (Year 1-1.5)
1	OD	0.16		0.16	0.32	0.4	0.4	8	4		
	OS*	0.4		0.63	0.63	0.63	0.63	3	1A	A+	A+
2	OD	0.5		0.63	0.8	1.0	1.0				
	OS*	0.3		0.63	0.8	0.8	0.8	4	2	1A	A+
3	OD*	0.63	*0.63*	0.63	0.8	0.8	*0.8*	1	A+	A+	*A+*
	OS	0.8	*0.8*	0.40	0.8	0.8	*0.8*	3	A+		
4	OD	0.4		0.50	0.8	0.8	0.8	3	1A		
	OS*	0.63		0.63	0.8	0.8	0.8	3	A+	A+	A+
5	OD	0.2		0.63	0.63	0.5	0.8				
	OS*	0.5		1	1.25	1.25	0.8			A+	A+
UDVA = Uncorrected Distance Visual Acuity, CDVA = (Best) Corrected Distance Visual Acuity, CNVA = (Best) Corrected Near Visual Acuity, UNVA = Uncorrected Near Visual Acuity, OD = Right eye, OS = Left eye, IOL = Intraocular Lens, SML = Scharioth Macula Lens, D = Diopter, Preop = Preoperative													

**Fig. 2:**
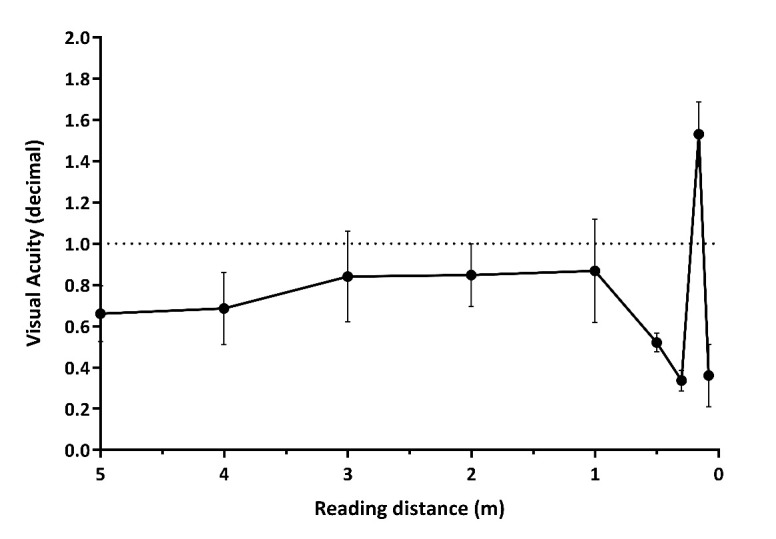
Binocular visual acuity curve (average of five patients) shows good visual acuity at all distances. Near vision is optimal at 15-18 cm reading distance

**Fig. 3:**
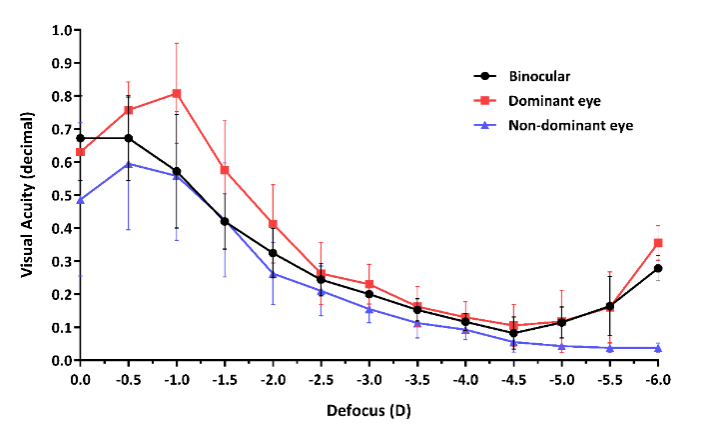
Monocular (dominant and non-dominant eye) and binocular defocus curves (average of five patients). The dominant eye ensures good far and near vision, while the non-dominant eye, targeted to slight myopia, provides appropriate intermediate vision

Binocular visual acuity curves and mono- and binocular defocus curves (the latter taken after distance correction) were plotted approximately one month postoperatively (**Fig. 2**,**3**). The visual acuity curve demonstrated that the best near visual acuity could be achieved at a reading distance of 16 cm (UNVA=1.53 ± 0.16; mean ± SD, expressed in decimal). Binocular distance visual acuity was also above 0.8 on the decimal scale in most of the cases (**Fig. 2**).

Defocus curve of the dominant eyes targeted for emmetropia, and thereafter implanted with the SML, showed two peaks of visual acuity: one for distance vision and one for near vision (**Fig. 3**). This distance corrected the near vision peak that could be seen with -6.0 D defocus addition, as that addition equaled the actual addition on the retinal plane provided by the SML. The fellow eye targeted for -1.0 D ensured good intermediate vision. Binocular vision is characterized by appropriate vision at all distances, however, near vision and distance vision will be the best with monovision of the patient.

Until the finishing of the manuscript, none of the patients have complained about either dysphotopic phenomena, or the loss of stereopsis in their daily activities.

## Discussion

The demand for spectacle independence and sharp vision at all distances is understandable also among patients suffering from high or excessive myopia. Several corneal or refractive surgical techniques have been widespread to correct the patients’ intense refractive error, however, none of them has brought visual outcomes without compromise [**5**-**19**]. The most obvious and safe solution seems to be the implantation of multifocal IOLs, but the near addition of these lenses is usually not enough to properly correct the near vision of high myopes: a remarkable residual refraction could be expected, and what also must be considered is that MIOLs are often not available in very low or even negative refractive powers [**15**]. Due to the unusual refractive power and lens thickness of these IOLs, the calculation of the optimal IOL-power also faces difficulties [**12**-**16**]. Moreover, MIOLs are reported to transfer only a reduced amount of light on to the retina, compared to monofocal lenses, which might affect the patients’ vision in low light conditions [**22**]. Additionally, because of multifocal optics, dysphotopic sensations after the MIOL-implantation are often reported [**22**]. All these considered, the implantation of MIOLs is usually contraindicated in patients with high or excessive myopia.

The new concept using the SML as a supplementary lens in patients with extreme or high myopia (26.0 mm < axial length AXL; 6.0 D < refractive error) seems to be an efficient approach in achieving appropriate vision at all distances, by creating a Binocular Trifocal Monovision.

Contrary to conventional multifocal intraocular lenses, in which the near addition is usually not high enough for extreme myopes to ensure proper near vision for reading without spectacles, the 2-fold magnification provided by the SML is able to bring real spectacle independence. Meanwhile, the distance vision corrected by the intracapsular IOL of the same eye remains unaffected, and the intermediate vision created by the implantation of the monofocal capsular bag IOL with slightly myopic target into the fellow eye has also proven to be comfortable for the patient. Both the binocular visual acuity curve and the defocus curves (monocular and binocular) reflect the patient’s subjective assessment: good visual outcome can be achieved throughout a wide range of distance. However, the optimal reading distance is around 15 cm, similarly as it was expected, and as it is shown in the case of AMD-patients implanted with the SML [**20**]. The defocus curves confirm the efficiency of the Binocular Trifocal Monovision concept: the emmetropia-targeted eye supplemented with the SML represents an appropriate magnification for reading and other activities requiring near vision and improved distance vision at the same time, while the mild myopic eye ensures good intermediate vision.

As this novel approach means the implantation of two monofocal primary IOLs and a SML, the disadvantages, like light energy loss and dysphotopic phenomena, deriving from the multifocal optic, can be avoided, which consequently improve the patient’s quality of vision. Nevertheless, targeted examinations are required to justify this assumption.

The five patients, whose results were introduced in this current paper, however, supported the above proposition, as they all welcomed the visual outcome with high satisfaction. During the postoperative period, neither any adverse events, complications or dysphotopic visual disturbances were experienced, hence the approach of achieving appropriate uncorrected vision at all distances with three intraocular lenses does not only seem to be an efficient, but also a safe method to treat high or excessive myopia.

Our current favorable results with this novel management of high myopia require further evaluation indeed, but our initial success encourages us to prove the safety and efficacy of the Binocular Trifocal Monovision concept with the enrolment of further patients suffering from high or excessive myopia.

## Conclusion

*What was known*

• Several approaches for the improvement of visual quality of patients with high or extreme myopia are known, however, the clinical outcomes of traditional methods are often either difficult to predict, or are unsatisfactory for the patient because of compromised vision and/ or side effects. 

• Multifocal IOLs are usually contraindicated for high or extreme myopes, as surgical outcomes are difficult to predict, and near vision cannot be properly corrected with the ordinary additions provided by most MIOLs on the market.

*What this paper adds*

• The concept of Binocular Trifocal Monovision achieved by the well-designed binocular implantation of monofocal capsular bag IOLs supplemented with a SML into the ciliary sulcus of the emmetropia-targeted eye seems to be a safe and promising approach in the visual correction of patients with high or extreme myopia.

• Appropriate uncorrected vision could be achieved at all distances; patients welcomed the visual outcome with high satisfaction.

• Adaption to the reduced reading distance did not cause any difficulty. None of the patients have complained either about disturbing dysphotopic phenomena, or about the loss of stereopsis.

**Conflict of Interest statement**

The authors state no conflict of interest.

**Informed Consent and Human and Animal Rights statement**

Informed consent has been obtained from all individuals included in this study.

**Authorization for the use of human subjects**

Ethical approval: The research related to human use complies with all the relevant national regulations, institutional policies, is in accordance with the tenets of the Helsinki Declaration, and has been approved by the review board of Aurelios Augenzentrum Recklinghausen, Recklinghausen, Germany.

**Acknowledgements**

None.

**Sources of Funding**

None.

**Disclosures**

Dr. Scharioth is a consultant to Alcon Laboratories, Inc., and Medicontur Ltd. He is the inventor of the Scharioth Macula Lens and receives royalties.
